# Shafarenko M, Tonfighi T, eds. Essential Med Notes 2019. Comprehensive medical reference & review for USMLE II and MCCQE. 35th edition. Toronto Notes for Medical Students, Thieme; 2019. ISBN:978-1-927363-49-2

**DOI:** 10.3325/cmj.2019.60.391

**Published:** 2019-08

**Authors:** Marina Raguž

**Field of medicine:** General medicine, medical education.

**Audience:** Senior medical students engaged in clinical rotations and preparing licensing exams (United States Medical Licensing Examination II and Medical Council of Canada Qualifying Examination), junior general and emergency medicine practitioners, residents, and other interested physicians.

**Purpose**: The book provides an overview of the basic knowledge in all fields of clinical medicine, including anatomy, physiology, pathology, diagnostics, clinical presentation, and therapy. The content is presented through text, pictures, tables, graphs, and explanatory text paragraphs. The wealth of information makes this book a useful source for all interested readers.

**Content:** Each field of clinical medicine is represented by one of the thirty three alphabetically organized chapters. Each chapter consists of a basic anatomy and physiology review, diagnostics, pathology, and clinical presentation of the diseases, differential diagnosis and therapy, followed by a list of references.

The first two chapters consist of tables containing *Common Unit Conversions* and *Commonly Measured Laboratory Values*, and the third chapter *Ethical, Legal and Organizational Medicine* discusses ethical and legal issues in medicine, such as confidentiality, consent, capacity, end-of-life care, etc.

The chapters focused on internal medicine and related subspecialist fields (*Cardiology, Endocrinology, Gastroenterology, Hematology, Nephrology, Respirology, Rheumatology*) all begin with the basic anatomical overview of the particular organ system, differential diagnosis of common clinical presentations, and routine diagnostic tests and medications. Further on, each chapter focuses on the specific diseases under separate sections, for example, cardiac arrhythmias, ischemic heart disease, heart failure, disorders of glucose metabolism, pituitary gland disorders, anemias, hematologic and lymphoid malignancies, diseases of the gastrointestinal and hepatobiliary systems, acute and chronic kidney injuries, pulmonary vascular diseases, respiratory failure, septic arthritis, osteoarthritis, etc.

Ten chapters related to surgical disciplines and anesthesia (*Anesthesia and Perioperative Medicine, Cardiac Surgery, General Surgery, Thoracic Surgery, Ophthalmology, Orthopedics, Otolaryngology, Plastic Surgery, Urology, Vascular Surgery*) also begin with an introductory section. For anesthesia, the types of anesthesia and preoperative assessment are first reviewed. In the chapters related to surgical disciplines, introduction contains the basic review of the anatomy of the respective system, differential diagnosis of common presentations, preoperative preparations, general procedures, common surgical complications, emergencies, and routinely used medications. Specific diseases are discussed in separate sections, such as cardiac transplantation, bowel obstruction, appendix surgery, breast surgery, fractures, facial nerve paralysis, brachial plexus conditions and surgical treatment, craniofacial injury, urinary trauma, peripheral venous diseases, carotid stenosis. Pediatric surgery is described in a special section of each chapter.

Three chapters related to the nervous system, *Neurology*, *Neurosurgery and Psychiatry*, also include an introductory section containing basic anatomical review, differential diagnosis, neurological and psychiatric assessment, routinely used medications, diagnostic tests, imaging methods, and different neurosurgical approaches. Specific diseases, such as seizure disorders and epilepsy, behavioral neurology, mild traumatic brain injury, neurooncology, movement and cerebellar disorders, tumors, abscesses, cerebrovascular diseases, vascular malformations, back pain, extradural lesions, neurotrauma, pediatric and functional neurosurgery, as well as psychotic disorders, mood disorders and anxiety, trauma and stress related disorders, are thoroughly discussed in separate sections.

*Gynecology* and *Obstetrics* chapters, after a basic overview, focus on disorders of menstruation, endometriosis, pregnancy, infertility, oncology, multi-fetal gestation and malpresentations, hypertension, labor and delivery, and puerperal care. Chapter *Pediatrics* merges together all clinical fields (internal medicine, surgical disciplines, infectious diseases, and neonatology).

*Emergency Medicine, Family Medicine,* and *Geriatric Medicine* are three separate chapters describing specific patient assessment in each field, and focus on major principles, common clinical presentations, and therapeutic management related to each of these disciplines.

The chapters *Infectious Diseases* and *Dermatology* after introductory general review describe specific conditions, such as gastrointestinal infections, travelers' disease, infections of the heart and CNS, systemic infections, tuberculosis, HIV, fever of unknown origin, heritable disorders, and malignant skin conditions.

The chapter *Medical Imaging* contains description of specific imaging modalities for each organ system, whereas chapter *Medical Genetics* starts with the introduction to genetics, and then focuses on specific genetic syndromes and diseases. Chapter *Clinical Pharmacology* reviews general principles of pharmacokinetics, pharmacodynamics, drug reactions, therapeutic responses, and interactions.

The chapter *Public Health and Preventive Medicine* describes the methodological tools for determining and measuring the population health, epidemiology, study designs, methods of data analysis, research methods, epidemiology of infectious diseases, and environmental and occupational health.

**Highlights:** The book contains all essential information and provides an excellent overview of the various clinical conditions. Lateral page border contains concluding remarks, which help the reader to repeat and summarize the provided information. The comprehensive index at the end of the book enables quick orientation and selective reading. The text is accompanied by numerous schematic presentations, radiological images, and photographs, which make learning both easy and exciting. Additional materials, *Clinical Handbook, Essential Medical Notes, and Handouts* (approach to ECG containing examples, cardiac condition algorithms, and ophthalmology test materials), are useful tools for everyday clinical work.

**Related reading**: A number of other comprehensive medical references either in book or pocket-size handbook format are available: Oxford Medical Handbook, Oxford University Press, 10th Edition, 2017 (for emergency medicine, anesthesia, internal medicine, clinical and health care research, clinical and laboratory research, clinical pharmacology and pathology, surgery etc.); Harrison’s Principles of Internal Medicine, McGraw-Hill Education, 20th Edition, 2018; Principles and Practice of Surgery, Elsevier, 6th Edition, 2012; Clinical Cases and OSCEs in Surgery, Elsevier, 3rd Edition, 2017; USMLE Step 2 CK Lecture Notes, Kaplan Medical, 1st Edition, 2019: 5-book set; First Aid for the USMLE Step 2 CK, McGraw-Hill Education, 10th Edition, 2019.

